# The association between family history of hypertension and diabetic kidney disease in patients with diabetes: a cross-sectional study

**DOI:** 10.3389/fendo.2026.1774744

**Published:** 2026-03-09

**Authors:** Yuwei Xing, Xusheng Yang, Qianqian Zhao

**Affiliations:** 1 Department of Endocrinology, The Second Hospital of Shijiazhuang, Shijiazhuang, China; 2 Department of Nephrology, Beijing Rehabilitation Hospital, Affiliated to Capital Medical University, Beijing, China

**Keywords:** family history, hypertension, kidney, propensity score matching, type 2 diabetes

## Abstract

**Introduction:**

The contribution of a family history of hypertension (HH) to diabetic kidney disease (DKD) pathogenesis remains unclear. Investigating this association is crucial for refining risk stratification and enabling early intervention in patients with type 2 diabetes (T2D). This study examined whether a HH is associated with higher odds of DKD among individuals with T2D

**Methods:**

Participants with T2D were grouped according to the presence or absence of a family history of hypertension. Propensity score matching (PSM) was used to mitigate potential confounding factors from baseline clinical features across the comparative groups. To evaluate robustness against confounding and missingness, we applied multivariable logistic regression and E-value analysis and then performed PSM on the dataset after multiple imputation.

**Results:**

The final analytical data comprised 1,612 individuals fulfilling the diagnostic criteria for T2D, comprising 1,419 without HH and 193 with HH. After PSM, 386 patients (193 patients per group) were included. PSM analysis yielded an odds ratio (OR) of 2.57 (95% CI: 1.48–4.46, *P* = 0.001). Similar estimates were obtained using inverse probability of treatment weighting (OR = 2.18, 95% CI: 1.52–3.13, *P* < 0.001) and other weighting approaches. The key findings maintained statistical significance throughout sensitivity testing.

**Discussion:**

In this cross-sectional study, a history of familial hypertension was significantly associated with higher odds of DKD in individuals with T2D. Given the cross-sectional design, causality and temporal direction cannot be established; prospective studies are needed to determine whether familial hypertension contributes to DKD development and progression.

## Background

Diabetic kidney disease (DKD), a leading cause of end-stage renal disease(ESRD) worldwide, shows strong familial aggregation. While family history of diabetes is a well-established risk factor for DKD, growing evidence indicates that a family history of hypertension(HH) independently increases DKD risk and improves risk stratification when incorporated into predictive models (1–3). Mechanistically, the genetic predisposition to hypertension and the excessive activation of the renin-angiotensin-aldosterone system (RAAS) can lead to the production of more NADPH oxidase and mitochondrial-derived reactive oxygen species (ROS) in diabetic glomeruli, the activation of TGF-β, and abnormal fibrous formation, thereby resulting in earlier and more severe podocyte loss, mesangial expansion, and tubulointerstitial fibrosis (4–6). Together, these epidemiologic and mechanistic data support systematic collection of family histories of both hypertension and diabetes in clinical risk assessment and early screening.

Therefore, this research project sought to evaluate the association between familial hypertension and a higher prevalence of DKD among individuals with clinically diagnosed type 2 diabetes (T2D). Using propensity score matching(PSM), we aimed to provide robust evidence on this relationship by minimizing confounding biases. These findings are expected to offer critical insights into refining risk stratification and informing early screening strategies for DKD.

## Materials and methods

### Study population

**Figure 1:**
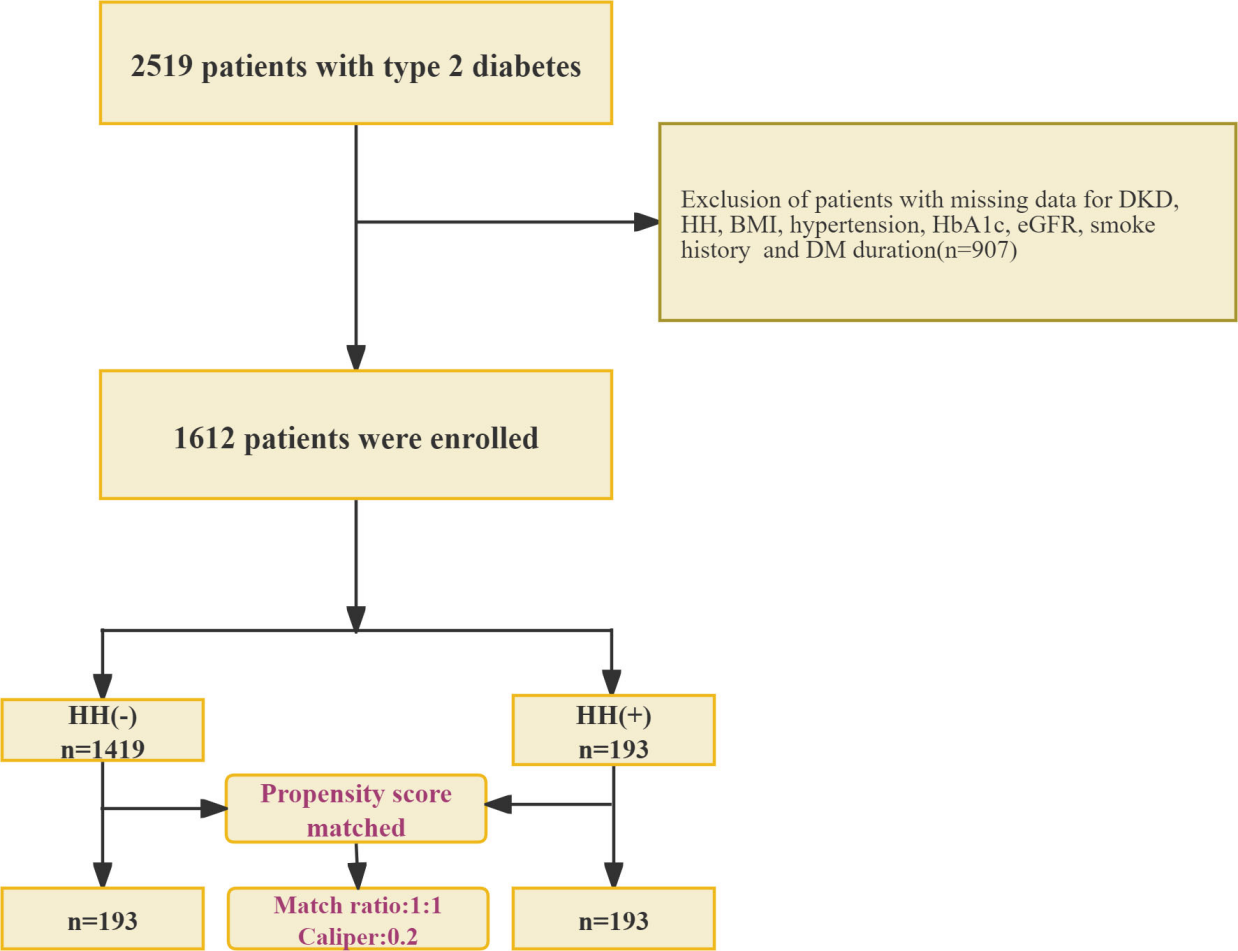
Patients enrollment flowchart. This flowchart illustrates the patient selection process for investigating the association between a family history of hypertension (HH) and DKD. HH, Family History of Hypertension; DM, Type 2 Diabetes; DKD, diabetic kidney disease; BMI, Body Mass Index; HbA1c, Glycated Hemoglobin; eGFR, Estimated Glomerular Filtration Rate. The formation of two comparison groups (n=193 each) suggests a propensity score matching or case-control design to ensure comparability.

We conducted a cross-sectional study of 2,519 subjects receiving care at the Department of Endocrinology, Second Hospital of Shijiazhuang City, Hebei Province, from January 2024 to January 2025 as the research subjects (**Figure 1**). The system collects and organizes the general clinical data of patients, covering indicators such as sex, age, smoking history, height, weight, duration of diabetes, and concurrent hypertension. The participants’ body weight and height were recorded while they wore light indoor clothing and were barefoot. A calibrated electronic column scale (Omron, China) was used to obtain weight measurements that were accurate to 0.1 kg. Height was determined to the nearest 0.1 cm by employing a wall-mounted stadiometer. Subsequently, the BMI was derived from the formula: weight (in kilograms) divided by height (in meters) squared (kg/m²).

This study strictly adhered to the guidelines of the Declaration of Helsinki and was approved by the Ethics Review Committee of the Second Hospital of Shijiazhuang City (Ethics Approval number:SEY202011117). Because the study was retrospective, and all participant data were fully anonymized, the ethics committee waived the requirement for informed consent.

Participant recruitment adhered rigorously to the World Health Organization (WHO) 1999 established diagnostic criteria for diabetes (7). Individuals were classified as having diabetes if they fulfilled one or more of the following items: characteristic symptoms of diabetes accompanied by a random venous plasma glucose level ≥ 11.1 mmol/L; a fasting state plasma glucose concentration ≥ 7.0 mmol/L; or a plasma glucose value ≥ 11.1 mmol/L measured 2 hours after a 75-g oral glucose tolerance test (OGTT). Confirmatory testing on a separate day is mandatory for asymptomatic individuals. Exclusion criteria were type 1 diabetes, gestational diabetes, other specific types of diabetes, acute diabetic complications, concurrent urinary tract infection, hematuria (including during menstruation), non-diabetic renal disease, ESRD requiring dialysis, hematological diseases, and malignancy.

### Laboratory biochemical indices

Venous blood specimens were obtained from each participant the morning after they had fasted for a minimum period of 8 h overnight. The analyzed biochemical parameters included glycated hemoglobin (HbA1c), fasting blood glucose (FBG), postprandial blood glucose (PBG), and serum creatinine (sCr). In addition, first-morning urine samples were obtained from each participant on three consecutive days for the measurement of urinary microalbumin, urinary creatinine, and the urinary albumin-to-creatinine ratio (UACR), which was subsequently calculated from these values.

### Definition of DKD

The diagnosis of DKD was not based on a single laboratory measurement. To align with standard clinical diagnostic criteria requiring evidence of persistent abnormality, we performed a retrospective review of electronic health records for all participants. Data from a minimum period of 12 months preceding study enrollment were examined. DKD case status was assigned if the record met any of the following criteria (8):

1. Persistent albuminuria: Two or more UACR measurements ≥30 mg/g, separated by at least three months.
2. or Progressive eGFR decline: eGFR (<60 mL/min/1.73 m²) for over 3 months.
3. or A pre-existing clinical diagnosis: A formal diagnosis of “DKD” recorded by the treating physician, which inherently implies the application of sustained abnormality criteria.

### Definition of family history

The family history of hypertension was simplified into a binary variable: if the respondent reported that at least one of their father, mother, or siblings had been diagnosed with hypertension by a doctor, it was classified as “yes”; otherwise, it was classified as “no” (9).

### Statistical analysis

Continuous data are reported as mean ± standard deviation or as median and interquartile range (IQR), depending on their distribution. Categorical data are represented as frequencies and percentages. For comparisons across groups, Student’s t-test was applied to normally distributed continuous variables, whereas the Mann-Whitney U test was used for non-normally distributed continuous data, and the chi-square test for categorical variables.

Among the 2,519 initially screened patients with T2D, 907 were excluded because of missing data for key variables, including DKD status, HH, BMI, hypertension status, HbA1c, eGFR, smoking history, and duration of diabetes. Consequently, 1,612 patients were included in the final analysis.

To balance between-group confounders to reduce the effect of potential bias as much as possible, we used PSM (10). PSM was used in this cross-sectional analysis solely as a robust technique to reduce measured confounding and to improve comparability of participant characteristics between groups. Because exposure and outcome were assessed concurrently, PSM was applied for covariate adjustment and not to imply causal inference or to emulate a randomized controlled trial.We performed 1:1 nearest-neighbor matching without replacement on the logit of the propensity score (caliper width = 0.2 standard deviations) to create matched data of 193 HH[-] patients paired with 193 HH[+] patients. The evaluation of covariate balance both prior to and following PSM was conducted using standardized mean differences (SMD) visualized using a love plot (formerly lover plot).

**Figure 2:**
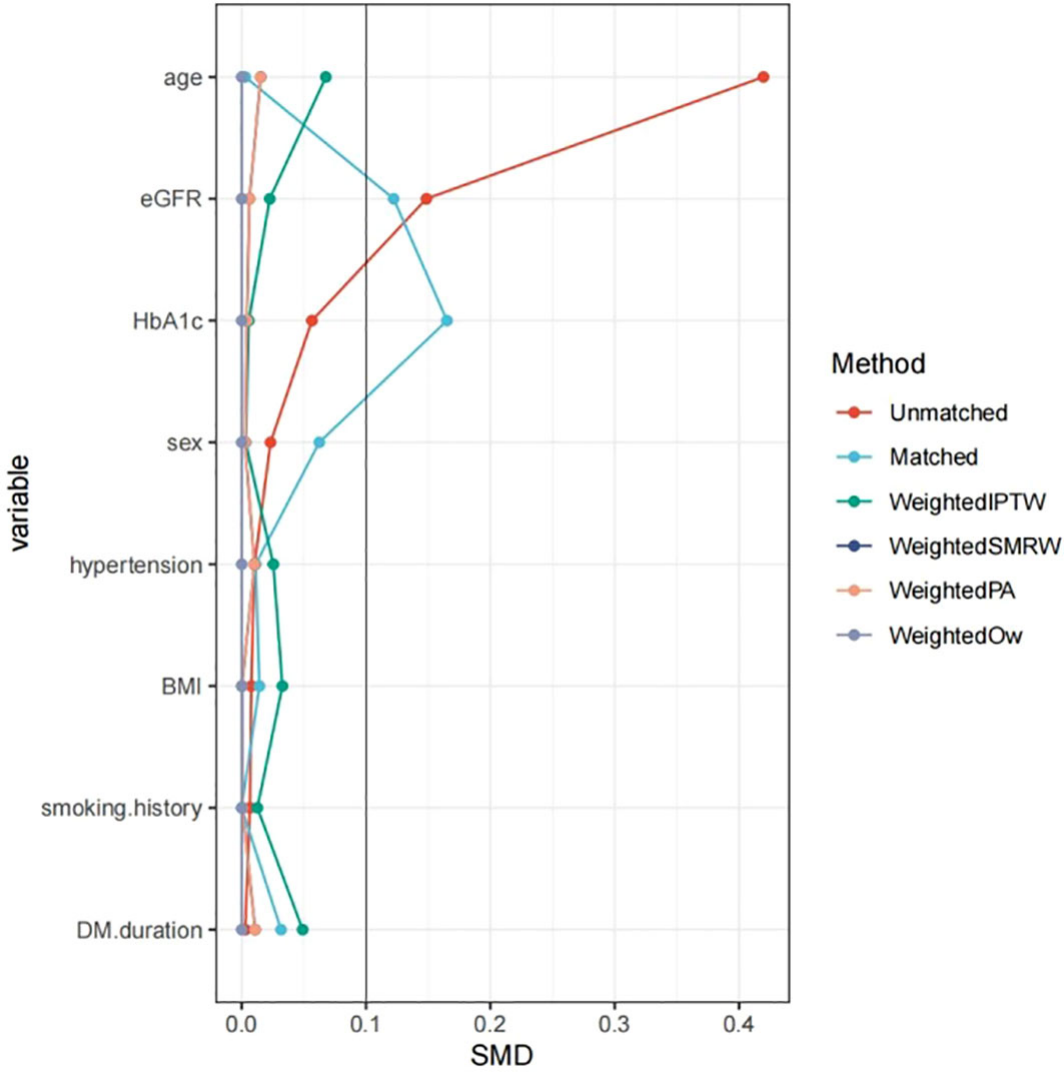
Family history of hypertension and DKD after PSM. BMI, Body Mass Index; DKD, diabetic kidney disease; HbA1c, Glycated Hemoglobin; eGFR, Estimated Glomerular Filtration Rate; SMD, Standardized Mean Difference; IPTW, Inverse Probability of Treatment Weighting; SMRW, Standardized Mortality Ratio Weighting; PA, Propensity Adjustment; OW, Overlap Weighting. Notes: This Love plot visually compares the absolute Standardized Mean Differences (SMDs) for baseline covariates across different adjustment methods. The vertical dashed line at SMD = 0.1 represents the commonly accepted threshold for adequate balance. Covariates with an SMD below this line after adjustment were considered well-balanced between the compared groups. The applied methods include unmatched (crude) comparison, propensity score matching (Matched), and various weighted approaches (IPTW, SMRW, PA, and OW).

Propensity scores were derived from logistic regression models that incorporated demographic and key clinical covariates. To assess the robustness of the findings, we applied multiple propensity score weighting techniques including Inverse Probability of Treatment Weighting (IPTW), Standardized Mortality Ratio Weighting (SMRW), Pairwise Algorithmic Weighting (PA), and Overlap Weighting (OW). An SMD value below 0.1 was considered indicative of acceptable covariate balance. Based on the estimated propensity scores as weights, an inverse probability weighting (IPW) model was employed to create weighted data (11). After 1:1 PSM, most covariates were well-balanced with standardized mean differences <0.1. However, modest residual imbalance remained for HbA1c (SMD = 0.165) and eGFR (SMD = 0.122) (**Figure 2**). These variables were therefore further adjusted for in a doubly robust logistic regression model to minimize potential confounding.

To assess robustness, several sensitivity analyses were performed. First, to prevent the loss of information during the matching process, we fitted a multivariate logistic regression model in the completely unmatched sample and adjusted for the same covariates as those included in the propensity score. We estimated three hierarchical models (1): unadjusted (2), adjusted for demographic characteristics, BMI, hypertension, and smoking, and (3) adjusted for HbA1c, eGFR, and duration of diabetes. Second, to examine whether the deletion of observations with missing values introduced bias and to verify the result stability through multiple imputation. The missing covariate data were processed using the multiple imputation by chained equations (MICE) method, generating five imputed datasets. To achieve a single and reproducible PSM process, these five imputed datasets were combined into a complete analysis dataset by averaging the imputed values (with a sample size of 2,519 cases). Then, PSM was performed using the complete analysis dataset along with inverse probability of treatment weights (IPTW, to estimate the average treatment effect), standardized mortality rate weights (SMRW, to evaluate the effect of the exposed/treated population), and overlap weights (emphasizing the covariate balance region). Because one variable (diabetes duration) remained imbalanced across groups after MICE+PSM, we conducted a doubly robust regression.

Finally, E-values were calculated for the main effect estimates to quantify the minimum intensity of unmeasured confounding factors on the risk ratio(RR) scale, and the level of intensity of such factors needs to be reached to explain the observed association. All sensitivity models were adjusted for the same covariates as in the propensity score model.

Data analysis was conducted using R Statistical Software (http://www.R-project.org, The R Foundation) and Free Statistics software version 2.3 (12). A two-sided p-value below 0.05 was defined as the threshold for statistical significance.

## Result

### Baseline characteristics

We analyzed 1,612 patients (**Figure 1**); 11.97% had a HH and 14.7% had DKD. **Table 1** summarizes the baseline characteristics: participants with a positive family history were younger, demonstrated a higher eGFR, and showed a greater prevalence of DKD. After 1:1 PSM, the analytic sample comprised of 386 patients (193 per group). Except for HbA1c and eGFR, the SMD for all other covariates were below 0.1 (**Table 1**).

**Table 1:** Comparison of baseline characteristics before and after propensity score matching.

Characteristic	Before matching					After matching				
	Total (n = 1612)	HH(-)	HH(+)	SMD	*P*-value	Total (n = 386)	HH (–)	HH(+)	SMD	*P*-value
		(n=1419)	N=193				(n=193)	(n=193)		
Demographic Characteristics										
Sex, n(%)		660 (46.5)	92 (47.7)	0.023	0.762		86 (44.6)	92 (47.7)	0.062	0.54
Male	860 (53.3)	759 (53.5)	101 (52.3)			208 (53.9)	107 (55.4)	101 (52.3)		
Female	752 (46.7)	660 (46.5)	92 (47.7)			178 (46.1)	86 (44.6)	92 (47.7)		
Age, years, Mean ± SD	63.2 ± 11.2	63.7 ± 11.1	59.1 ± 11.1	*0.42*	< 0.001	59.1 ± 11.0	59.0 ± 10.9	59.1 ± 11.1		0.978
Anthropometric Measures										
BMI, kg/m^2^, Mean ± SD	26.0 ± 6.3	26.0 ± 6.5	26.0 ± 4.3	0.008	0.93	26.1 ± 4.1	26.1 ± 4.0	26.0 ± 4.3	0.014	0.889
Medical History										
DM duration, years, Median (IQR)	2.0 (1.0, 6.0)	2.0 (1.0, 6.0)	3.0 (1.0, 6.0)	0.003	0.933	2.0 (1.0, 6.0)	2.0 (1.0, 7.0)	3.0 (1.0, 6.0)	0.032	0.966
Hypertension, n (%)				0.01	0.892				0.011	0.912
No	486 (30.1)	427 (30.1)	59 (30.6)				8.90 (2.07)	8.56 (2.03)		
Yes	1126 (69.9)	992 (69.9)	134 (69.4)				127.88 (54.29)	121.52 (49.61)		
Lifestyle Factors										
Smoking history, n (%)				0.007	0.931				<0.001	1
No	1358 (84.2)	1195 (84.2)	163 (84.5)			326 (84.5)	163 (84.5)	163 (84.5)		
Yes	254 (15.8)	224 (15.8)	30 (15.5)			60 (15.5)	30 (15.5)	30 (15.5)		
Laboratory Parameters										
HbA1c, %, Mean ± SD	8.5 ± 1.9	8.5 ± 1.9	8.6 ± 2.0	0.057	0.452	8.7 ± 2.1	8.9 ± 2.1	8.6 ± 2.0	*0.165*	0.106
eGFR, mL/min/1.73 m2, Mean ± SD	115.1 ± 48.1	114.3 ± 47.9	121.5 ± 49.6	*0.149*	0.05	124.7 ± 52.0	127.9 ± 54.3	121.5 ± 49.6	*0.122*	0.231
Complications										
Diabetic kidney disease (DKD), n (%)					< 0.001					< 0.001
No	1375 (85.3)	1230 (86.7)	145 (75.1)			316 (81.9)	171 (88.6)	145 (75.1)		
Yes	237 (14.7)	189 (13.3)	48 (24.9)			70 (18.1)	22 (11.4)	48 (24.9)		

BMI, body mass index; DM, diabetes mellitus; eGFR, estimated glomerular filtration rate; HbA1c, glycated hemoglobin; HH(+), family history of hypertension positive; HH (–), family history of hypertension negative; n, umber; SD, standard deviation; SMD, standardized mean difference.

Data are presented as mean (SD) for continuous variables and n (%) for categorical variables.

Propensity score matching was performed in a 1:1 ratio using the nearest-neighbor method without replacement, with a caliper width of 0.2.

### The association between HH and DKD: results after PSM

As shown in **Table 2**, a HH was associated with DKD across all analytical approaches. The unmatched crude OR was 2.15 (95% CI: 1.50–3.09, *P* < 0.001). Following multivariate adjustment for clinical covariates, the association remained significant (OR = 2.30, 95% CI: 1.56–3.40, *P* < 0.001). PSM yielded an OR of 2.57 (95% CI: 1.48–4.46, *P* = 0.001), and all weighting methods demonstrated consistent effect estimates: IPTW (OR = 2.18, 95% CI: 1.52–3.13), SMRW (OR = 2.10, 95% CI: 1.46–3.01), PA (OR = 2.10, 95% CI: 1.24–3.54), and OW (OR = 2.10, 95% CI: 1.19–3.69). This association persisted in the doubly robust analysis (IPTW combined with outcome regression adjusting for HbA1c and eGFR), yielding an adjusted OR of 2.66 (95% CI 1.49–4.73; *P* = 0.001) (**Table 2**).

**Table 2:** The association between family history of hypertension and DKD: Results after propensity score matching.

Models	OR (95%CI)	*P* value
Unmatched.crude	2.15 (1.5~3.09)	<0.001
PropensityScore.adjusted	2.1 (1.46~3.03)	<0.001
PropensityScore.Matched	2.57 (1.48~4.46)	0.001
Weighted.IPTW	2.18 (1.52~3.13)	<0.001
Weighted.SMRW	2.1 (1.46~3.01)	<0.001
Weighted.PA	2.1 (1.24~3.54)	0.006
Weighted.Ow	2.1 (1.19~3.69)	0.01
Doubly robust analysis	2.66(1.49~4.73)	0.001

OR, Odds Ratio; CI, Confidence Interval; DKD, diabetic kidney disease; IPTW, Inverse Probability of Treatment Weighting; SMRW, Standardized Mortality Ratio Weighting; PA, Precision Weighting; OW, Overlap Weighting.

### Sensitive analysis

To preserve the initial sample and probe the stability of the principal findings, multivariable regression was performed on the completely unmatched data as a sensitivity analysis. In the unadjusted analysis (Model 1), the OR was 2.15 (OR = 2.15, 95% CI: 1.5–3.09, *P* < 0.001). Following control for demographic and lifestyle covariates (Model 2), the odds ratio was 2.16 (95% CI 1.48–3.12; *P* < 0.001); additional adjustment for metabolic parameters in Model 3 yielded an OR of 2.3 (95% CI 1.45–3.4; *P* < 0.001) (**Table 3**).

**Table 3:** Association between family history of hypertension and DKD in patients with type 2 diabetes.

HH	Total	n.event%	Model 1		Model 2		Model 3	
			Crude.OR (95%CI)	Crude.*P* value	adj.OR (95%CI)	adj.*P* value	adj.OR (95%CI)	adj.*P* value
No	1419	189 (13.3)	1(Ref)		1(Ref)		1(Ref)	
Yes	193	48 (24.9)	2.15 (1.5~3.09)	<0.001	2.15 (1.48~3.12)	<0.001	2.3 (1.56~3.4)	<0.001

HH, Family history of hypertension; DKD, diabetic kidney disease; OR, Odds Ratio; CI, Confidence Interval; Ref, reference.

Model 1 is unadjusted.

Model 2 is adjusted for sex, age, body mass index (BMI), hypertension, and smoking history.

Model 3 is adjusted for all variables in Model 2 plus glycated hemoglobin (HbA1c), estimated glomerular filtration rate (eGFR), and diabetes mellitus (DM) duration.

E-values showed that the observed association (RR 2.3, 95% CI 1.56–3.40) (**Supplementary Table 1**) would require an unmeasured confounder related to both exposure and outcome by a RR of at least 4.03 (lower 95% CI bound 2.49) to fully negate the effect. These results underscore the robustness of our findings to potential residual confounding factors. After imputation and PSM, crude, adjusted, and weighted analyses yielded ORs of 1.64–1.87 (*P ≤* 0.031) (**Supplementary Table 2**), indicating persisting positive associations in those models. After controlling for diabetes duration using a doubly robust approach, the association remained, with an adjusted OR of 1.58 (95% CI 1.04–2.41; P = 0.033) (**Supplementary Table 2**).

## Discussion

This study is one of the few to analyze the relationship between HH and DKD. After PSM, a family history of hypertension was associated with a higher prevalence of DKD. This conclusion was confirmed through logistic analysis and e-value as a sensitivity analysis. This indicates that even after considering a series of covariates such as the duration of diabetes, BMI, and hypertension comorbidities, the association between HH and DKD remains quite significant. Taken together, these findings suggest that the observed association between familial hypertension and DKD is unlikely to be spurious. However, it is crucial to interpret these results within the context of the study’s cross-sectional design. Our analysis identifies an independent association at a single point in time and cannot establish a causal or predictive relationship.

BMI, a simple anthropometric proxy for diet, physical activity and lifestyle, shows a dose-dependent association with blood pressure and hypertension (13). Mendelian-randomization indicates a causal role of excess adiposity on vascular outcomes largely mediated by higher systolic and diastolic blood pressure (14). Yet BMI can miss adverse fatness; body-composition and metabolic metrics improve individual risk assessment (15).

The investigation of familial hypertension as a determinant of DKD has been relatively limited compared to research on metabolic, clinical, and lifestyle risk factors. Existing evidence, however, suggests that a family history of hypertension constitutes an independent risk factor for DKD across diabetes types. Studies in both type 1 and type 2 diabetes have linked familial hypertension with the onset and progression of albuminuria and clinical DKD, supporting a role for inherited vascular susceptibility in renal injury (16–19). This association persists in diverse populations, including adolescents with type 1 diabetes (20), and individuals with diabetes post-kidney transplantation (21), suggesting shared genetic or environmental mechanisms underlying hypertension, diabetes, and kidney disease (22).

These studies have suggested an association between family history and specific diseases, which can serve as the basis for our research. However, the literature is hampered by studies that are outdated, limited to specific disease contexts, or that consider family history only partially as a risk determinant, preventing a thorough appraisal of its impact on DKD. Therefore, to clarify the contribution of family history, we evaluated the independent relationship between HH and DKD.

Genetic predisposition may disrupt glomerular hemodynamics, enhance tubular sodium reabsorption, impair vascular reactivity, and increase renal susceptibility. Concurrent metabolic disturbances, particularly insulin resistance and oxidative stress, can amplify endothelial dysfunction and tubular injury, providing a coherent biological pathway by which familial hypertension may promote DKD (23–25). Hannedouche et al. observed that patients with type 1 diabetes with a family history of hypertension exhibited early hyperfiltration and attenuated hemodynamic responses to angiotensin-converting enzyme inhibitors(ACE inhibitors), suggesting an intrinsic defect in renal vascular regulation that may predispose to later kidney damage and DKD progression (26). Additionally, the genetically influenced elevation of erythrocyte sodium–lithium countertransport, which marks salt sensitivity and a tendency toward hypertension, has been linked to higher odds of DKD, which is more pronounced in patients with hypertensive relatives, pointing to a heritable pathway from blood−pressure susceptibility to renal injury (27–29).

Studies have found that family history is associated with broader cardiovascular, kidney, and metabolic risks. Family history of hypertension is also associated with increased risk of other CKD-related outcomes, including metabolic syndrome and cardiovascular disease, further supporting a common familial risk profile (30, 31).

While our results suggest a potential link, they highlight the need for further prospective and mechanistic research to confirm this association. If future studies establish a causal relationship, these findings could support the consideration of a family history of hypertension as a more prominent element in risk stratification algorithms for DKD. This, in turn, might inform the development of targeted screening protocols and public health strategies aimed at early detection and intervention.

A principal strength of this study is the application of PSM to reduce measured confounding in a cross-sectional data. Using PSM, we observed a statistically significant association between a family history of hypertension and a higher prevalence of DKD among individuals with T2D. However, several constraints must be considered. First, the cross-sectional nature of our study prevents firm causal conclusions because the temporal relationship between exposure and outcomes cannot be determined. Because HH(exposure) and DKD(outcome) were measured at the same time point in this cross-sectional study, temporal ordering cannot be determined and causal inference is not supported. Although PSM improves covariate balance for measured confounders, it cannot address unmeasured or unknown confounding and does not substitute for prospective measurement or randomization. Therefore, our findings should be interpreted as associations that require confirmation in longitudinal or experimental studies to establish causal relationships. Second, information on family history was self-reported (restricted to physician-diagnosed hypertension) and, therefore, may be vulnerable to recall errors or misclassifications. Third, a sizable fraction of initially screened subjects were excluded for missing essential variables, which could introduce selection bias; although we performed multiple sensitivity analyses, this potential bias cannot be fully ruled out. Fourth, Although PSM achieved balance for most baseline characteristics, residual imbalance in HbA1c and eGFR persisted. While the magnitude of these differences was modest, we cannot entirely exclude the possibility of residual confounding. To mitigate this concern, we employed doubly robust estimation, which adjusts for these covariates directly in the outcome model. The consistency between the doubly robust estimates and the primary matched analysis supports the robustness of our findings. Fifth, we lacked detailed data on relatives’ age at hypertension onset, participants’ antihypertensive and glucose-lowering medication use (for example Angiotensin-converting enzyme inhibitors(ACE inhibitors)/angiotensin II receptor antagonists(ARBs)or sodium-glucose cotransporter 2 inhibitors (SGLT2 inhibitors)), baseline UACR, and the intensity/frequency of clinical testing, all factors that could affect DKD risk or its detection, and thus represent possible unmeasured confounders. Finally, because the data were drawn from a single ethnic group/setting, the findings require confirmation in other populations and prospective studies for broader generalizability.

## Conclusion

In this cross-sectional study, a history of familial hypertension was significantly associated with higher odds of DKD in individuals with T2D. Given the cross-sectional design, causality and temporal direction cannot be established; prospective studies are needed to determine whether familial hypertension contributes to DKD development and progression.

## Data Availability

The raw data supporting the conclusions of this article will be made available by the authors, without undue reservation.

## Supplementary material

Supplementary Figure 1 Family history of hypertension and DKD after PSM of multiple imputation data. BMI, Body Mass Index; DKD, diabetic kidney disease; HbA1c, Glycated Hemoglobin; eGFR, Estimated Glomerular Filtration Rate; SMD, Standardized Mean Difference; IPTW, Inverse Probability of Treatment Weighting; SMRW, Standardized Mortality Ratio Weighting; PA, Propensity Adjustment; OW, Overlap Weighting. This Love plot visually compares the absolute Standardized Mean Differences (SMDs) for baseline covariates across different adjustment methods. The vertical dashed line at SMD = 0.1 represents the commonly accepted threshold for adequate balance. Covariates with an SMD below this line after adjustment were considered well-balanced between the compared groups. The applied methods include unmatched (crude) comparison, propensity score matching (Matched), and various weighted approaches (IPTW, SMRW, PA, and OW).

Supplementary Table 1 E-value analysis for assessing the impact of potential unmeasured confounding. RR, risk ratio; CI, confidence interval.

Supplementary Table 2 The association between family history of hypertension and DKD: Results after propensity score matching of multiple imputation data. OR, Odds Ratio; CI, Confidence Interval; DKD, diabetic kidney disease; IPTW, Inverse Probability of Treatment Weighting; SMRW, Standardized Mortality Ratio Weighting; PA, Precision Weighting; OW, Overlap Weighting. * The multivariable model was adjusted for sex, age, BMI, hypertension, smoking.history, HbA1c, eGFR, DM.duration.
