# Supplementary figures and images for: The association between family history of hypertension and diabetic kidney disease in patients with diabetes: a cross-sectional study
Source: Front Endocrinol (Lausanne). 2026 Mar 9;17:1774744. doi: 10.3389/fendo.2026.1774744 (PMC13006290; doi:10.3389/fendo.2026.1774744)

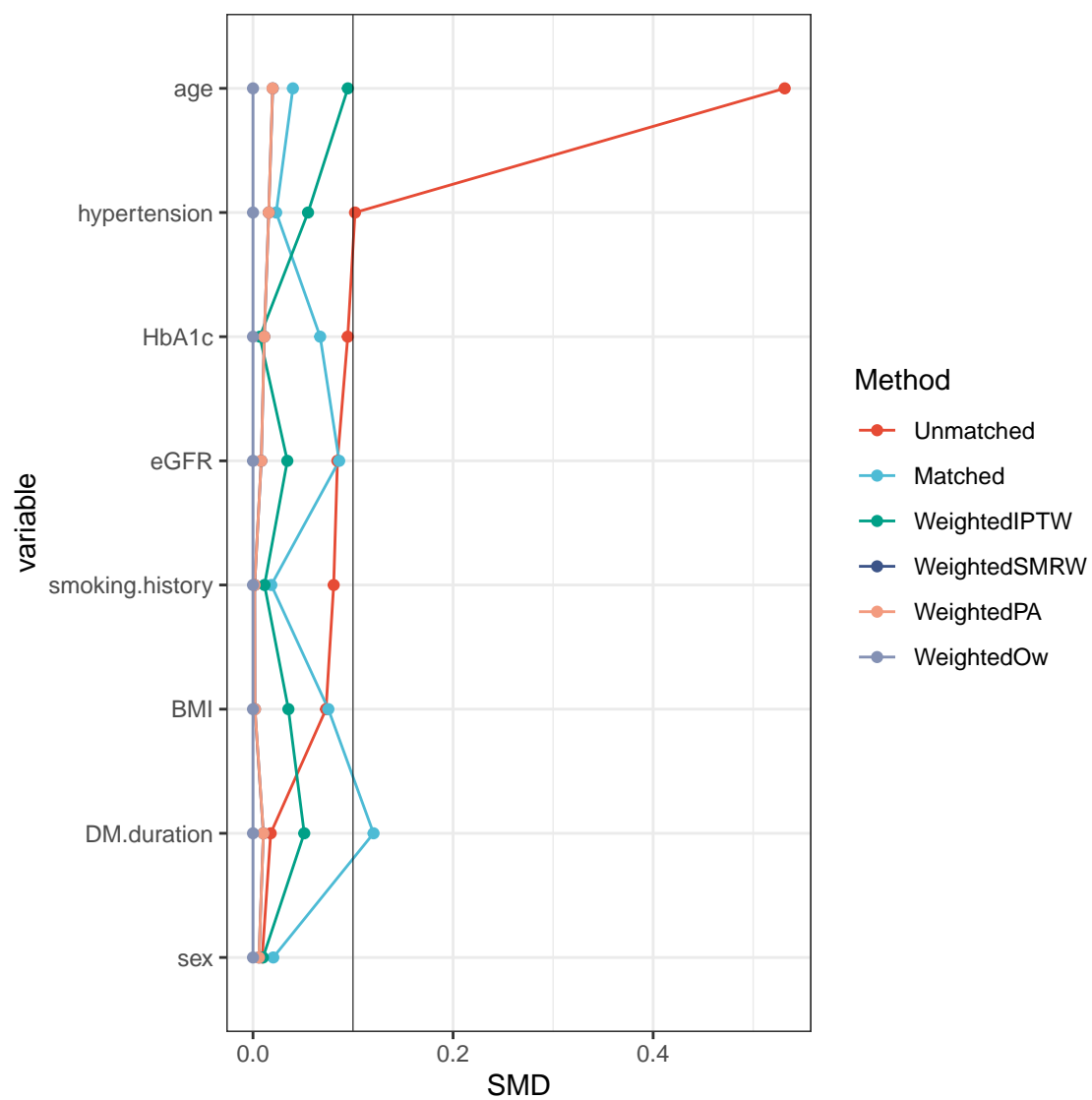

Supplement: Supplementary Figure 1 — Family history of hypertension and DKD after PSM of multiple imputation data. BMI, Body Mass Index; DKD, diabetic kidney disease; HbA1c, Glycated Hemoglobin; eGFR, Estimated Glomerular Filtration Rate; SMD, Standardized Mean Difference; IPTW, Inverse Probability of Treatment Weighting; SMRW, Standardized Mortality Ratio Weighting; PA, Propensity Adjustment; OW, Overlap Weighting. This Love plot visually compares the absolute Standardized Mean Differences (SMDs) for baseline covariates across different adjustment methods. The vertical dashed line at SMD = 0.1 represents the commonly accepted threshold for adequate balance. Covariates with an SMD below this line after adjustment were considered well-balanced between the compared groups. The applied methods include unmatched (crude) comparison, propensity score matching (Matched), and various weighted approaches (IPTW, SMRW, PA, and OW). [file DataSheet1.pdf]
