# Supplementary material for: The association between family history of hypertension and diabetic kidney disease in patients with diabetes: a cross-sectional study
Source: Front Endocrinol (Lausanne). 2026 Mar 9;17:1774744. doi: 10.3389/fendo.2026.1774744 (PMC13006290; doi:10.3389/fendo.2026.1774744)
Supplement: Supplementary Table 1 — E-value analysis for assessing the impact of potential unmeasured confounding. RR, risk ratio; CI, confidence interval. [file Table1.docx]

**Supplementary Table 1. E-value Analysis for Assessing the Impact of Potential Unmeasured Confounding**

| **Term** | **Value** |
| --- | --- |
| RR | 2.3 (95% CI: 1.56, 3.4) |
| E-value | 4.03(95% CI: 2.49, NA) |

Abbreviations: RR, risk ratio; CI, confidence interval.
