# Supplementary material for: The association between family history of hypertension and diabetic kidney disease in patients with diabetes: a cross-sectional study
Source: Front Endocrinol (Lausanne). 2026 Mar 9;17:1774744. doi: 10.3389/fendo.2026.1774744 (PMC13006290; doi:10.3389/fendo.2026.1774744)
Supplement: Supplementary Table 2 — The association between family history of hypertension and DKD: Results after propensity score matching of multiple imputation data. OR, Odds Ratio; CI, Confidence Interval; DKD, diabetic kidney disease; IPTW, Inverse Probability of Treatment Weighting; SMRW, Standardized Mortality Ratio Weighting; PA, Precision Weighting; OW, Overlap Weighting. * The multivariable model was adjusted for sex, age, BMI, hypertension, smoking.history, HbA1c, eGFR, DM.duration. [file Table2.docx]

**Supplemary Table 2.The association between family history of hypertension and DKD: Results after propensity score matching of multiple imputation data**

| **Analysis** | **OR (95%CI)** | ***P* value** |
| --- | --- | --- |
| Unmatched.crude | 1.87 (1.39~2.52) | <0.001 |
| Multivariable.adjusted* | 1.78 (1.28~2.46) | 0.001 |
| PropensityScore.adjusted | 1.68 (1.24~2.27) | 0.001 |
| PropensityScore.Matched | 1.44 (0.96~2.16) | 0.08 |
| Weighted.IPTW | 1.82 (1.35~2.46) | <0.001 |
| Weighted.SMRW | 1.64 (1.22~2.21) | 0.001 |
| Weighted.PA | 1.64 (1.08~2.49) | 0.02 |
| Weighted.Ow | 1.65 (1.05~2.6) | 0.031 |

Abbreviations: OR, Odds Ratio; CI, Confidence Interval; DKD, diabetic kidney disease; IPTW, Inverse Probability of Treatment Weighting; SMRW, Standardized Mortality Ratio Weighting; PA, Precision Weighting; OW, Overlap Weighting.

* The multivariable model was adjusted for sex，age，BMI，hypertension，smoking.history，HbA1c，eGFR，DM.duration.
